# Early Diagnosis of Cardiovascular Diseases in Workers: Role of Standard and Advanced Echocardiography

**DOI:** 10.1155/2018/7354691

**Published:** 2018-01-16

**Authors:** Lidia Capotosto, Francesco Massoni, Simone De Sio, Serafino Ricci, Antonio Vitarelli

**Affiliations:** Sapienza University, Rome, Italy

## Abstract

Cardiovascular disease (CVD) still remains the main cause of morbidity and mortality and consequently early diagnosis is of paramount importance. Working conditions can be regarded as an additional risk factor for CVD. Since different aspects of the job may affect vascular health differently, it is important to consider occupation from multiple perspectives to better assess occupational impacts on health. Standard echocardiography has several targets in the cardiac population, as the assessment of myocardial performance, valvular and/or congenital heart disease, and hemodynamics. Three-dimensional echocardiography gained attention recently as a viable clinical tool in assessing left ventricular (LV) and right ventricular (RV) function, volume, and shape. Two-dimensional (2DSTE) and, more recently, three-dimensional speckle tracking echocardiography (3DSTE) have also emerged as methods for detection of global and regional myocardial dysfunction in various cardiovascular diseases and applied to the diagnosis of subtle LV and RV dysfunction. Although these novel echocardiographic imaging modalities have advanced our understanding of LV and RV mechanics, overlapping patterns often show challenges that limit their clinical utility. This review will describe the current state of standard and advanced echocardiography in early detection (secondary prevention) of CVD and address future directions for this potentially important diagnostic strategy.

## 1. Introduction

Cardiovascular disease (CVD) still remains the main cause of morbidity and mortality [1, 2] and consequently early diagnosis is of paramount importance. Although the division of prevention of cardiovascular disease into primary, secondary, and tertiary prevention is arbitrary, it may be useful for development of services by different parts of the health care system. Working conditions can be regarded as an additional risk factor for CVD [3]. Since different aspects of the job may affect vascular health differently, it is important to consider occupation from multiple perspectives to better assess occupational impacts on health [4]. Standard echocardiography has several targets in the cardiac population, as the assessment of myocardial performance, valvular and/or congenital heart disease, and hemodynamics. Three-dimensional echocardiography gained attention recently as a viable clinical tool in assessing left ventricular (LV) and right ventricular (RV) function, volume, and shape. Two-dimensional (2DSTE) and, more recently, three-dimensional speckle tracking echocardiography (3DSTE) have also emerged as methods for detection of global and regional myocardial dysfunction in various cardiovascular diseases [5–9] and applied to the diagnosis of subtle LV and RV dysfunction. Although these novel echocardiographic imaging modalities have advanced our understanding of LV and RV mechanics, overlapping patterns often show challenges that limit their clinical utility. This review will describe the current state of standard and advanced echocardiography in early detection (secondary prevention) of CVD and address future directions for this potentially important diagnostic strategy.

## 2. Cardiovascular Disease in Workers

Atherosclerosis, the basis of coronary artery disease (CAD), develops into a complex process [10]. CAD is recognized as a paraoccupational disease; thus working conditions could be regarded as a possible risk factor for disease onset, development, or deterioration [11]. The relationships between psychosocial work load and CAD, as well as interactions among neuropsychological and immunological systems, have also attracted attention [12].

Although stress-related disorders comprise only a small fraction of occupational injuries and illnesses [11], overall the median time away from work is more than four times greater for stress-related conditions than for all other diseases. Work-related stress (WRS) is thought to contribute to several occupational illnesses including cardiovascular disease. All the cohort studies and most cross-sectional studies found greater carotid artery intima-media thickness (CIMT) with higher levels of WRS. A large body of literature showed a close relationship between WRS and CAD [13–24] as well as a relationship between occupational stress and early atherosclerotic changes found in carotid ultrasound examinations [25–29]. Differences in body mass index, systemic hypertension, and smoking across studies may explain some conflicting results [11] since selection of patients may be a challenge in interpreting occupational epidemiology data. Actually, workers in high strain jobs may have bad health behaviors that cannot be adequately adjusted for statistical models and act as confounding factors. Additionally, workers with known heart disease could choose jobs with lesser degrees of stress or leave overall their work, and people who work in high strain occupations who develop heart disease may potentially change their positions with new ones with lower degrees of stress. However, the triad of occupational exposures, such as shift work, noise, and physical workload [3], emerged as significant risk factor of CAD. Their joint effects did not show any high risk peaks, but the relative risk of those with all three risk factors present was about twofold compared with those with none of the factors. In another study [20], in comparison with daytime-only workers, rotating shift workers reported higher job strain, exhibited flatter *α*-amylase and cortisol diurnal slopes, reduced daily *α*-amylase production, elevated daily cortisol production, and reduced heart rate variability and endothelial functioning.

A deleterious effect of shift work was also shown on lipid metabolism [21–24]. Women working in a rapid forward rotating shift pattern had poorer sleep quality according to self-reported indicators of the validated Pittsburgh Sleep Quality Index and they had a higher prevalence of the metabolic syndrome compared with women working during the day only [21]. Working shift was, independently of lifestyle or BMI, significantly related to more elevated plasma triglycerides and rate of hypertriglyceridemia, lower plasma HDL-C levels, and hypertension compared to nonshift daytime work [22]. The prevalence of lifestyle-related risk factors including hypertension, diabetes, dyslipidemia, metabolic syndrome, and obesity was higher in men than women and higher overall in workers aged 60–69 years [23], reinforcing the importance of developing effective strategies for the prevention of cardiovascular disease among middle-aged and older workers, especially in men.

Many features of the environment exert an important influence on CVD risk, progression, and severity. Numerous epidemiological and experimental studies showed that air pollution [25, 26] causes a systemic vascular oxidative stress reaction. Increased cardiovascular mortality was also related to long-term and short-term exposure to nitrogen dioxide. Exposure to air pollution and road traffic was associated with an increased risk of arteriosclerosis with premature aortic and coronary calcification and increased risk of systemic hypertension, myocardial infarction, stroke, and acute heart failure. The Global Burden of Disease Study 2015 ranked ambient exposure to fine particulate matter with an aerodynamic diameter of <2.5 *μ*m (PM_2.5_) as the fifth most important risk factor for mortality worldwide and the burden from air pollution was comparable to that from hypertension and diabetes mellitus [25]; thus it should be considered an important modifiable environmental cardiovascular risk factor.

Whereas the link between occupation and cardiovascular disease has been recognized, it is unclear which role occupation plays in the progression of subclinical CVD measured as carotid intima-media thickness (CIMT). Few longitudinal studies exist about occupation and subclinical CVD. Furthermore often these studies do not examine whether occupation is only an indicator of the person's socioeconomic position or a source of potentially health-compromising exposures. While in the former approach occupational differences show the socioeconomic gradient in CVD, in the latter approach occupation represents additional damage to the risk profile.

A weak positive correlation was found between CIMT and age, diastolic blood pressure, LDL cholesterol, and antiphospholipid antibodies, and a negative correlation with the presence of anti-cardiolipin antibodies and a general level of occupational stress [26–28]. A more complicated association was shown between occupational mobility and CIMT [29]. Specifically, for those patients with lower initial occupational standings, upward mobility was associated with less CIMT and downward mobility with greater CIMT, whereas for those with higher initial standings, upward mobility was associated with greater CIMT while downward mobility was unrelated to either CIMT measure.

## 3. Role of Echocardiography in Early Diagnosis of Specific CVD

### 3.1. Coronary Heart Disease

Coronary artery disease or ischemic heart disease (CAD or IHD) is one of the major causes of morbidity and mortality. Echocardiography provides a thorough complex assessment of structural and hemodynamic changes induced by acute or chronic CAD [30–32], and in skilled hands it is useful for the clinical management of these patients. Despite its undeniable operator dependence, its availability, ease of use, price, capacity to serve as bedside technique, and repeatability make the method essential for all cardiologists.

**Figure 1:**
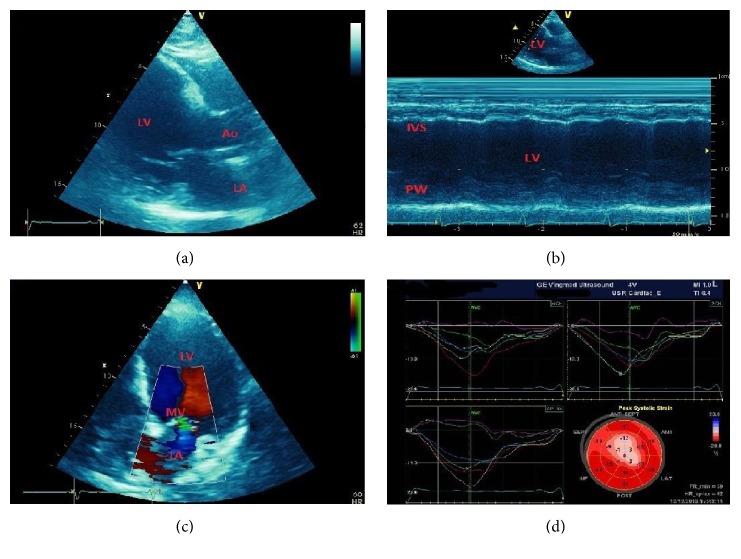
A 43-year-old male patient with anteroapical myocardial infarction. Subsequent stent implantation in the left anterior descending (LAD) coronary artery and medical therapy. (a) Dilated left ventricle (LV) in long axis view. (b) Akinetic scarred septum (IVS) in M-mode representation. (c) Dilated LV in apical 4-chamber view with mitral valve (MV) regurgitation. (d) White scarred segments compared to red normokinetic segments in the “bull's-eye” plot by speckle-tracking echocardiography. Strain curves show reduction of anteroapical longitudinal strain. Ao = aorta; LA = left atrium; PW = LV posterior wall.

Myocardial ischemia impairs regional systolic contractility. Persistence of severe ischemia produces development of myocardial necrosis and scarring with permanent functional impairment (Figure 1). Regional myocardial function is usually assessed only visually by assessing wall thickening and endocardial motion of myocardial segments, and is graded depending on the quality of contraction. The recommended scoring [32] is as follows: (1) normal or hyperkinetic, (2) hypokinetic (reduced thickening), (3) akinetic (absent or negligible thickening), and (4) dyskinetic (systolic thinning or stretching or aneurysm). Based on the segmental motion evaluation a wall motion score index (WMSI) can be calculated as a sum of all scores divided by the number of visualized segments. The segmentation of the left ventricular cavity is usually based on a 17-segments model.

The use of deformation (“strain”) echocardiography allows less subjective assessment of myocardial contraction as compared to simple visual evaluation [33–36]. The strain and strain rate derived from two-dimensional speckle tracking echocardiography (2DSTE) are based on computer algorithms tracking the movement of the so-called “speckles,” clusters of natural acoustic markers generated within the myocardium by an interaction with ultrasonic waves (Figure 1). Compared to Doppler techniques, this modality is independent of the ultrasound beam propagation angle and allows evaluation of longitudinal, radial, and circumferential strains.

Since in the normal myocardium strains and strain rates are homogenously distributed, subtle strain changes suggest myocardial contractile impairment. A good correlation has been shown between longitudinal strain decrease and reduced coronary perfusion in segments that appear visually normal. In addition to regional myocardial strain assessment, a global longitudinal strain (GLS) can be determined averaging peak longitudinal strain in all evaluable segments. GLS is compromised in CAD patients and appears as a useful index for assessment of global myocardial function. A GLS cut-point of greater than −17.77% had good sensitivity and specificity for detecting CAD and was comparable to a WMSI ≥ 1.13 measured during stress [34]. Unfortunately there is a disparity in results depending on the software and the vendor used. Thus the current recommendations on chamber quantification suggest normal values of GLS for a healthy individual around 20% but studies are in progress and routine clinical application of myocardial strain is not yet reached.

Three-dimensional speckle tracking echocardiography (3DSTE) has recently been regarded as a more promising technique to accurately and reproducibly evaluate the segmental and global LV function [36]. The 3D mode avoids foreshortening of apical views, consumes less time in data acquisition, helps to solve the problem of out-of-plane motion present in the 2D modality tracking motion of speckles in all three dimensions, needs one single apical 4-chamber view to carry out all the analysis, and has good reproducibility as automated method as shown by the lower intraobserver and interobserver variability. However, this advantage is achieved at the expense of lower frame/volume rate, lower temporal resolution, and higher dependence on image quality with possible inappropriate tracking due to dropouts in the endocardial border in patients with poor acoustic windows.

**Figure 2:**
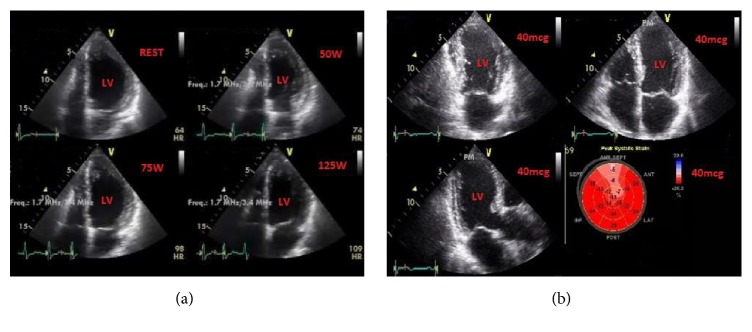
Provocative testing in ischemic heart disease. (a) Exercise stress echocardiography in a patient with normal coronary arteries. Persistent normal wall motion after exercise (125 W). (b) Dobutamine stress echocardiography in a patient with ischemic heart disease. Anteroseptal hypokinesia at peak dose (40 mcg) is shown both by wall motion abnormalities (top panels) and strain analysis (bottom right panel, lighter segments in the “bull's-eye” representation of peak systolic strain). LV = left ventricle.

The role of 2D stress echocardiography (SE), either exercise (exercise SE) or pharmacologic with dobutamine (DSE), is well established for diagnosis and prognosis of patients with known or suspected CAD [7, 37–52]. Appropriate uses of exercise SE (Figure 2) are low pretest probability of CAD and ECG uninterpretable or unable to exercise and high or intermediate pretest probability of CAD regardless of ECG interpretability and ability to exercise. An imaging stress test is also recommended in symptomatic patients with prior revascularization, to assess the functional severity of intermediate lesions on coronary arteriography, and as the initial test for diagnosing CAD if the left ventricular ejection fraction is <50% in patients without typical angina [40–42]. The dobutamine stress test (DSE) should be carried out when an exercise test cannot be performed.

The sensitivity of SE is higher for multivessel CAD and lower for single vessel disease. Sensitivity of the test is also influenced by the time between peak stress and image acquisition. It is imperative to accomplish postexercise imaging as soon as possible (≤1 min from cessation of exercise). When abnormalities recover rapidly, false-negative results occur. On the other hand, despite the excellent specificity of SE, some patients have false-positive tests (i.e., <50% diameter coronary artery stenosis on the subsequent angiogram) in the absence of left bundle branch morphology, right ventricular pacing, prior cardiac surgery, or abnormal wall tethering at baseline. These false-positive findings present a management challenge because it remains unclear whether these patients should be treated and how to treat them. A subset of patients has microvascular abnormalities [43], a hypertensive response to exercise, vasomotor changes, endothelial dysfunction, and/or small vessel coronary disease that can lead to false-positive SE.

2D-SE has its own limitations as multiple views of the left ventricle (LV) must be obtained from more than window to completely visualize all segments. Three-dimensional stress echocardiography (3D-SE) allows assessing overall wall motion of the entire LV simultaneously in different planes [44]. 3D images can be displayed in multiplane or multislice views for ease of comparison with greater accuracy and interobserver agreement when compared to 2D. 3D-SE is quantitative, provides rapid image acquisition, avoids LV foreshortening by correct alignment of imaging planes, and is easily applied during pharmacologic stress and feasible during exercise-induced stress. Despite these advantages, 3D has lower temporal and spatial resolution than 2D and requires longer analysis time.

A major limitation of echocardiographic study interpretation is the subjective visual analysis of endocardial motion and wall thickening which is only semiquantitative. The need for more quantitative techniques to objectively evaluate LV regional wall motion during DSE has led to the incorporation of new indices in the analysis of regional wall motion. Tissue Doppler imaging (TDI) is a novel echocardiographic technique that can be used to quantitatively assess low-velocity motion of myocardial walls with excellent temporal resolution [45]. 2D-strain echocardiography (2DSTE) is a highly sensitive alternative method of quantifying regional myocardial performance on the basis of grayscale ultrasound imaging. Several experimental studies have already validated 2D strain techniques against sonomicrometry during dobutamine infusion and/or ischemia. Clinical studies have investigated the diagnostic value of 2D strain and related parameters during DSE for inducible ischemia detection in patients with suspected CAD [7, 46, 47]. Two-dimensional STE is feasible on full-protocol DSE after ST-segment elevation myocardial infarction, provides incremental value to conventional visual wall analysis, and represents a promising new quantitative technique to detect significant angiographic CAD at follow-up (Figure 2).

Regional LV akinesia in CAD patients is irreversible in the presence of scarred tissue but may be reversible in stunned or hibernated myocardium [48–52]. Stunning and hibernation are different in terms of pathophysiology and clinical course but have similar prognostic implications related to the degree of LV dysfunction. Identification of viable myocardium becomes of critical importance in patients with LV ejection fraction <40% and high risk of mortality who may benefit from any improvement of myocardial contractility.

Assessment of contractile response to inotropic stimulation by low-dose dobutamine echocardiography or evaluation of myocyte metabolism with nuclear scan and positron emission tomography is used to identify hibernating myocardium. Recent clinical reports such as STICH trial suggest that we should not rely on one single imaging parameter and that optimal medical therapy may be sometimes as effective as revascularization procedures [49, 50]. However, although on the basis of the STICH trial there is no evidence that assessment of myocardial viability should not be included in the workup of patients with chronic LV dysfunction, the role of cardiac imaging in clinical decision-making could benefit from further studies.

### 3.2. Hypertensive Heart Disease

**Figure 3:**
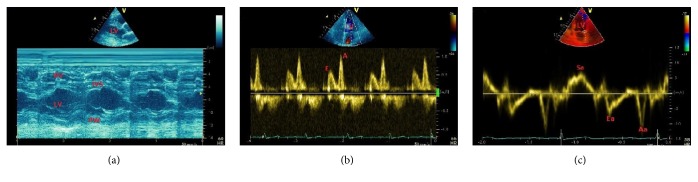
A 48-year-old male patient with systemic arterial hypertension, left ventricular hypertrophy, and diastolic dysfunction. Patient was on medical therapy. (a) Concentric left ventricular hypertrophy. (b) Mitral inflow velocity with inverted *E*/*A* ratio. (c) Mitral annulus velocity with inverted *E*_*a*_/*A*_*a*_ ratio. *E*_*a*_ reduction with increased *E*/*E*_*a*_ ratio suggests diastolic dysfunction. *A* = inflow late diastolic (atrial) velocity; *E* = inflow early diastolic velocity; *E*_*a*_ = annular early diastolic velocity; IVS = interventricular septum; LV = left ventricle; PW = LV posterior wall; RV = right ventricle; *S*_*a*_ = annular systolic velocity.

Hypertensive heart disease represents a form of organ damage with strong independent prognostic significance. Assessment of myocardial anatomy and function is necessary to identify early cardiac alterations in hypertensive patients since left ventricular hypertrophy (LVH) is the first step toward the development of coronary heart disease, stroke, heart failure, and sudden death. Recent Guidelines of the American College of Cardiology/American Heart Association [53] define normal blood pressure as <120/80 mmHg, elevated blood pressure 120–129/80 mmHg, hypertension stage 1 >130/80 mmHg, and hypertension stage 2 ≥140/90 mmHg and include echocardiography among the recommended techniques to assess the presence of preclinical organ damage in hypertensive patients (Figure 3). Although the relationship between baseline LV mass and incidence of cardiovascular events is independent of other cardiovascular risk factors, LV mass reproducibility is a major technical limitation of echocardiography since its calculation assuming a prolate ellipsoid shape may be not reliable in patients with asymmetrical hypertrophy or previous myocardial infarction. Observational studies as the LIFE [54] examined the potential clinical benefits of regression of echocardiographic detectable LVH during treatment, with important prognostic significance. The LIFE study confirmed the prognostic influence of LV geometry in addition to changes in LV mass and the association of a concentric geometry during treatment to a greater incidence of cardiovascular events. Three-dimensional echocardiography provided better correlations than 2D echo with cardiac magnetic resonance (CMR) measurements [55].

Echocardiography may also give useful information on cardiac functional performance, including systolic and diastolic changes (Figure 3). LV diastole may be examined by assessment of transmitral flow velocities, mitral annular pulsed tissue Doppler imaging, and left atrial volume [56]. Three-dimensional echocardiography improves the accuracy of the evaluation of left ventricular and left atrial volumes by eliminating the need for geometric modelling. Global LV longitudinal strain is a clinically useful parameter to identify subclinical systolic myocardial dysfunction [57]. In addition, LV segmental and global diastolic strain rate and diastolic untwist may be used to assess relaxation and diastolic function. Thus, 3D echocardiography, speckle tracking echocardiography, and CMR are the most promising methods to provide accurate assessment of cardiac structure and function for early detection of preclinical organ damage.

**Figure 4:**
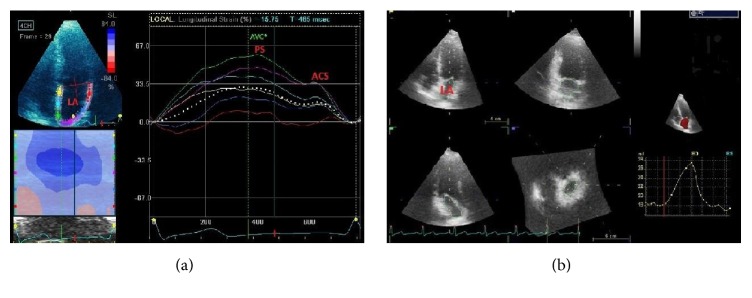
Left atrial (LA) size and function in systemic arterial hypertension. Novel technologies for LA analysis. (a) LA wall deformation curve by speckle tracking echocardiography. (b) LA volume by three-dimensional echocardiography. ACS = atrial contraction strain; PS = peak strain (peak global longitudinal strain at ventricular end-systole).

Systemic hypertension may be a potential independent risk factor for heart failure with preserved ejection fraction, and LV diastolic filling changes can be observed even in the absence of LV remodeling. Since the left atrium (LA) is directly exposed to LV pressures during ventricular diastole, LA size is correlated with LV filling pressure. Thus, LA structural remodeling represents a stable indicator of the severity of LV diastolic dysfunction [58–61]. Besides LA structure, assessment of LA function is a further step toward early diagnosis of abnormal LA-LV coupling (Figure 4). The development of two-dimensional speckle-tracking echocardiography (2DSTE) has facilitated the early detection of LA and LV dysfunction in the absence of LA dilatation or impaired LV relaxation and in the presence of preserved LV ejection fraction [62]. LV diastolic and systolic dysfunction was shown to be a potent independent predictor of LA structural and functional changes in asymptomatic patients with hypertension. The use of 2DSTE gave new insights into alterations in LA structure and function related to LV dysfunction, underlining the key role of LA-LV coupling.

The role of arterial stiffness in systemic hypertension has also been demonstrated and is based on its pathophysiological importance for overall cardiovascular performance [63–68]. The European guidelines for arterial hypertension suggested aortic pulse wave velocity as a tool for assessment of subclinical target organ damage [63]. Large artery stiffening increases LV afterload and is associated with LVH and impaired coronary perfusion [66]. Moreover, stiffening of large arteries is involved in the pathogenesis of hypertension [67, 68]. Aortic stiffness was shown to have a predictive value independent of classic cardiovascular risk factors and other potential confounders and to integrate the effect of the genetic background and the cumulative damage of risk factors on the arterial wall. The risk associated with increased arterial stiffness is similar to the risk of established risk predictors commonly used in clinical practice, such as LVH. Furthermore, it is a powerful predictor of all-cause mortality in addition to cardiovascular outcomes.

### 3.3. Heart Failure

Heart failure (HF) is a common clinical syndrome, especially in the elderly, but its diagnosis is often missed. A detailed clinical history is crucial and should address not only current signs and symptoms of heart failure but also those signs pointing to a specific cause of the syndrome, such as coronary artery disease, hypertensive heart disease, or valvular heart disease. Echocardiographic imaging has several targets in the HF population, including the assessment of myocardial structure and function, valvular disease, and hemodynamics.

All modalities of echocardiography are useful in the assessment of the HF patient [69–73]. Historically, M-mode echocardiography was the first technique, and it remains helpful for accurate assessment of wall thickness and chamber dimensions. The use of two-dimensional (2D) echocardiography has enhanced the assessment of LV volumes and valvular disease. Three-dimensional (3D) echocardiography has further improved the accuracy of measurements of chamber volumes and function and structural evaluation of regurgitation. Doppler assessment of hemodynamics in HF includes evaluation of pulmonary artery pressure, right atrial pressure, LV filling, and valvar regurgitation. Analysis of longitudinal and radial strain provides clinical applications in the assessment of subclinical ventricular dysfunction and dyssynchrony.

**Figure 5:**
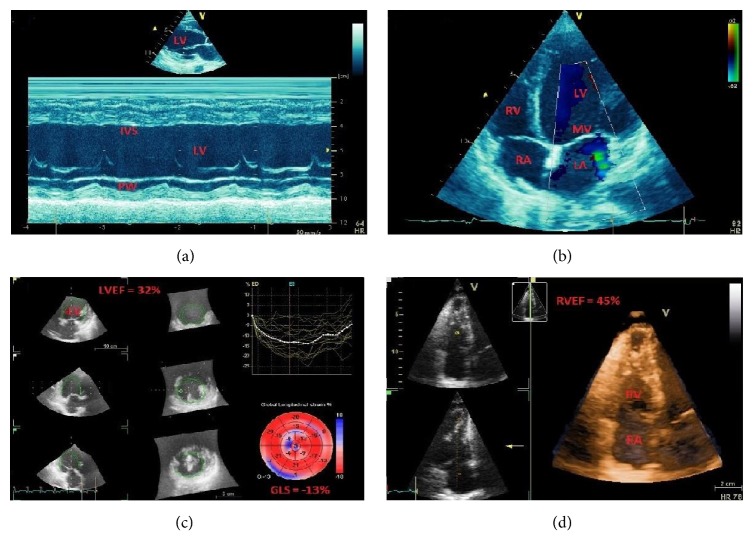
A 48-year-old male patient with dilated cardiomyopathy from previous myocarditis and systolic dysfunction. Patient was on medical therapy. (a) LV dilatation and septal (IVS) and posterior wall (PW) hypokinesia by M-mode echocardiography. (b) Two-dimensional four-chamber apical view showing LV end-systolic dilatation and mitral valve (MV) regurgitation. (c) Three-dimensional four-chamber apical view showing LV dilatation and severe systolic dysfunction (left ventricular ejection fraction = 32%). (d) Calculation of three-dimensional right ventricular volumes showing right ventricular ejection fraction at lower normal limits (45%). GLS = global longitudinal strain; IVS = interventricular septum; LV = left ventricle; LVEF = left ventricular ejection fraction; PW = LV posterior wall; RA = right atrium; RV = right ventricle; RVEF = right ventricular ejection fraction.

Two-dimensional echocardiographic assessment of LV ejection fraction is based on measuring end-diastolic and end-systolic volumes [32, 69–71] using different geometric models (length-diameter and length-area methods, Simpson's rule method, and modified Simpson's rule). These methods have shown good correlation with angiographic data for the assessment of left ventricular ejection fraction (LVEF), with normal LVEF generally considered to be ≥55–60%. Values below 50% are considered abnormal, and patients with LVEF ≤ 40% are considered to have significant systolic dysfunction (Figure 5) requiring initiation of specific medical therapy such as ACE-inhibitors, ß-blockers, and diuretics. Limitations of 2D-TTE include its operator-dependability and the inherent geometric assumptions of LV cavity. Three-dimensional echocardiography has emerged recently as a valid technique in assessing LV function, volume, and shape (Figure 5). Its ability to spatially define the cardiac structures in three-dimensional planes obviates the geometric assumptions necessary for 2D echocardiography. With further improvement, it has the potential to become an important complement for ejection fraction assessment.

Most HF patients have reduced LV systolic function; however a significant subset has normal or near normal resting systolic function with predominantly diastolic dysfunction [56, 74–77]. The distinction between systolic (LVEF < 35–40%) and diastolic (normal or elevated LVEF) dysfunction is critical for selecting proper therapies, since both entities may manifest clinically with indistinguishable signs and symptoms, but treatment and prognosis may be very different.

Diastolic dysfunction causes impairment of LV relaxation and compliance and consequent elevation of LV filling pressures, left atrial pressures, pulmonary venous pressures, pulmonary capillary pressures, and right heart pressures. Cardiac performance is compromised with exercise, even in the presence of normal stroke volume and cardiac output at rest. Doppler echocardiography can help in the diagnosis by allowing assessment of indices of diastolic filling and ventricular relaxation. Myocardial hypertrophy (Figure 3) and/or ischemia and myocardial fibrosis are the usual underlying pathologic processes for diastolic dysfunction and decreased ventricular compliance, as well as the normal aging process. Echocardiography can be used in cases of suspected restrictive pathology to differentiate constrictive pericarditis from infiltrative diseases such as myocardial amyloidosis and hemochromatosis. Tissue Doppler Imaging and Speckle Tracking Echocardiography may also be helpful [73].

The presence of RV dysfunction has important prognostic implications in patients with HF. RV dysfunction has often been evaluated qualitatively, but the development of RV annular systolic tissue velocity, RV free wall strain, and three-dimensional RV volumes (Figure 5) has been an improvement over existing conventional quantitative measures such as tricuspid annular displacement, fractional area change, and myocardial performance index [78, 79]. Survival, left ventricular ejection fraction and symptoms are worse in dilated cardiomyopathy patients with biventricular dysfunction (left ventricular ejection fraction < 50%, right ventricular ejection fraction < 35%) compared with those with LV dysfunction alone.

Selection of patients for a wide range of device therapies needs also an accurate echocardiographic evaluation [80–84]. These approaches concern cardioverter defibrillators implantation and cardiac resynchronization therapy (CRT) in the initial phases of dilated cardiomyopathy, while echocardiography may help in the decision of implantation of LV and RV assist devices in advanced HF. The use of CRT has been justified by prognostic benefit in HF patients with wide QRS, but functional benefit is not uniform in these patients. Actually, defining the response to CRT is not easy, and the concept of nonresponsiveness is still controversial [71, 80].

### 3.4. Congenital Heart Disease

Adults with congenital heart disease (CHD) have increased in number due to advances in early diagnosis and treatment [85]. Cardiovascular imaging is important in the long-term management of adult congenital heart disease (ACHD) because of persistent residual or postoperative right- and left-sided anatomic and haemodynamic abnormalities. Since symptoms may appear late, periodic imaging monitoring is essential to detect haemodynamic changes [86] and assess comorbidity including acquired heart disease that can occur with age [87]. The choice of the imaging modality is suggested by the lesion-specific characteristics of patients, advantages and limits of the technique, institutional resources, and expertise of the cardiologist. A multimodality imaging approach is often required to obtain all the necessary structural and functional information for the management of the postoperative patient [88–90].

Apart from the assessment of postoperative complex CHD, nowadays echocardiography has gained an important role in the management of patients with patent foramen ovale (PFO) and atrial septal defect (ASD). Strokes have high rates of morbidity being the second leading cause of death, and up to 40% of ischaemic strokes are cryptogenic. A strong association has been shown between cryptogenic stroke and PFO prevalence suggesting paradoxical embolism via PFO as a potential cause. PFO closure with Amplatzer device appeared superior to medical therapy in preventing strokes in patients with cryptogenic embolism [91], and the percutaneous transcatheter closure has become a well-established technique for the treatment of PFO and ASD up to a diameter of 35 mm [92].

PFO represents a remnant of the foetal foramen ovale and has a flap valve mechanism that intermittently opens a small channel between left and right atrium. It is associated with an intermittent right-to-left shunt, only rarely with a permanent left-to-right shunt, has an incidence of 20% to 35% of the general population, and is commonly treatable by device closure. On the contrary, ASD is a permanent opening characterized by left-to-right shunting with transient right-to-left shunting on the basis of the breathing cycle and accounts for nearly 10% of congenital heart disease. Different types of ASD involve particular structures such as septum primum (ostium primum ASD), septum secundum (ostium secundum ASD), sinus venosus (sinus venosus ASD), and coronary sinus (coronary sinus ASD). The ostium secundum ASD is the most frequent type and the only one treatable by percutaneous transcatheter closure. Indications for ASD closure are isolated secundum ASD with a pulmonary/systemic flow (*Q*p/*Q*s) ratio ≥1.5 : 1 and signs of right ventricular volume overload. Indications for PFO closure are cryptogenic stroke and evidence of right to left shunt.

**Figure 6:**
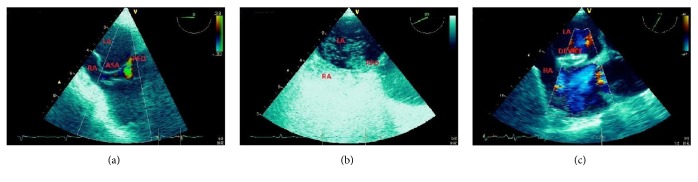
A 27-year-old male patient with patent foramen ovale (PFO) and a history of transient ischaemic attack (TIA). Patient had atrial device implantation. (a) Atrial septal aneurysm (ASA) with PFO and left-to-right shunt by transesophageal echocardiography. (b) TEE right-to-left shunt after intravenous injection of ultrasound contrast agent. (c) Postdevice transesophageal longitudinal bicaval view showing absence of shunt flow. LA = left atrium; RA = right atrium.

Transesophageal echocardiography (TEE) has become an established approach (Figure 6) to guide the interventional treatment of interatrial shunts [93–97]. Contrast transthoracic echocardiography (TTE) has a role as a screening tool in patients with unclear right volume overload or after systemic embolic events [98] but is not capable of differentiating between PFO and the various kinds of ASDs and not suitable for guiding percutaneous device closure of interatrial communications.

**Figure 7:**
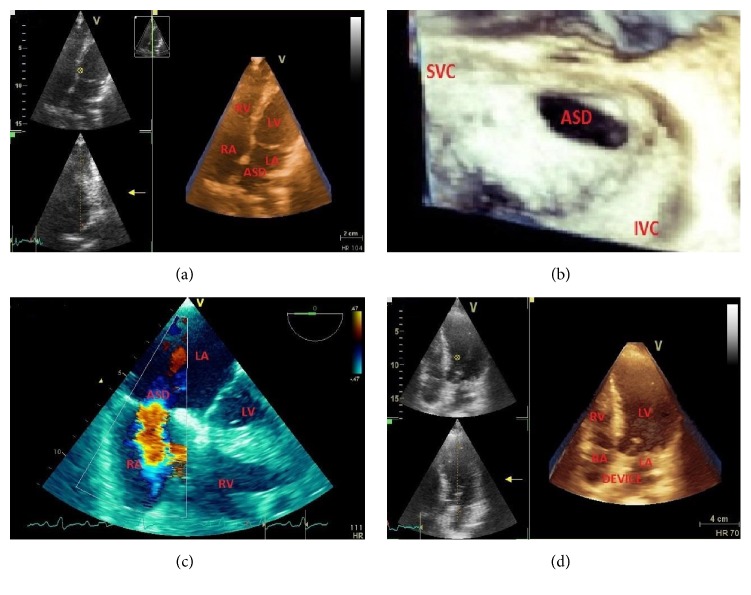
A 32-year-old female patient with ostium secundum atrial septal defect (ASD) and a history of ischaemic stroke. Patient had atrial device implantation. (a) Three-dimensional apical 4-chamber view, ASD. (b) Transesophageal three-dimensional longitudinal view, ASD shape. (c) Transesophageal 4-chamber view, left-to-right shunt by color Doppler. (d) Postdevice three-dimensional apical 4-chamber view showing absence of shunt flow. IVC = inferior vena cava; LA = left atrium; LV = left ventricle; RA = right atrium; RV = right ventricle; SVC = superior vena cava.

Traditionally, percutaneous ASD and PFO closures are performed under 2D-TEE guidance. TEE is also employed prior to device closure to assess defect size, position, and any associated left-to-right and right-to left shunting (Figure 7). It is important to assess the ASD rims of the respective structures: superior vena cava rim (superior), aortic rim (anterosuperior), coronary sinus rim (anteroinferior), IVC rim (inferior), and posterior LA rim (posterior). If the inferior rim length is <5 mm, the occluder closure is not indicated. Absence of the aortic rim is not a contraindication for the percutaneous closure but requires some degree of oversizing of the device encompassing the aortic root.

Life-threatening acute congestive LV failure complicating surgical or transcatheter closure of ASDs in patients with LV restriction has been reported [99, 100]. Device closure is an alternative to surgery in these patients, only on condition that there is no right-to-left shunting, LA pressure is markedly reduced due to echocardiography-guided preconditioning, LA pressure remains below the critical level of 20 mmHg on the temporary ASD closure, and the interventional cardiologist is familiar with the procedure and can complete it in a straightforward manner under echocardiographic guidance [96].

Three-dimensional TEE (3D-TEE) offers improved spatial orientation and better definition of ASD, “complex” PFO, or multifenestrated septal aneurysm [101, 102]. The major advantage of 3D-TEE is its capability of showing the dynamic morphology of ASDs with complex geometries, including elliptical, oblong, or fenestrated shapes. The technique is also used for preinterventional assessment of the atrial lesion and detection of complications, such as device dislodgement, device malposition, haemorrhagic pericardial effusion, device thrombosis, failure, or erosion.

### 3.5. Valvular Heart Disease

Patients with valvular heart disease (VHD) can be asymptomatic or present with a series of symptoms not always related to the severity of the lesion [103]. Current guidelines [104, 105] recommend transthoracic echocardiography (TTE) as the initial diagnostic test in patients with known or suspected VHD. While 2D and M-mode TTE allow assessment of valvular morphology, Doppler provides information on haemodynamics, pressure gradients, valve area, pulmonary artery pressure, and LV filling [106, 107]. Transoesophageal echocardiography (TEE) gives helpful detailed structural and hemodynamic information. Exercise Doppler echocardiography is useful to assess the patient's functional capacity and the impact of exercise on valvular and ventricular function [108–110]. Overall, the severity of a valvular lesion is better determined on the basis of a multiparameter echo Doppler assessment.

**Figure 8:**
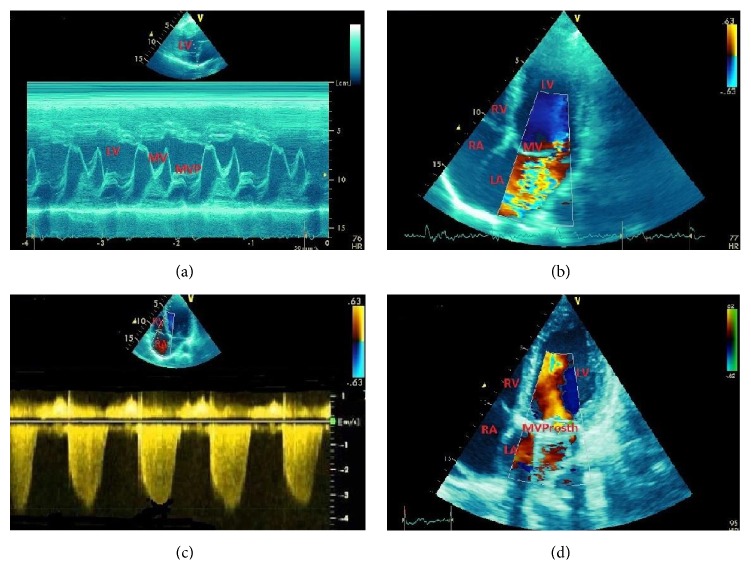
A 38-year-old male patient with congenital mitral valve prolapse (MVP), mitral regurgitation (MR), and progressive dyspnea. Patient had mitral valve surgery. (a) MVP by M-mode echocardiography. (b) Color Doppler in apical 4-chamber view showing severe MR. (c) Doppler-estimated systolic pulmonary artery pressure (58 mmHg). (d) MR reduction after mitral valve surgery (MV mechanical prosthesis). LA = left atrium; LV = left ventricle; MV = mitral valve; RA = right atrium; RV = right ventricle.

Mitral regurgitation (MR) is the most common clinically recognizable valvular heart condition. The disease has various congenital and acquired etiologies. Mitral valve prolapse (MVP) is a primary condition (Figure 8) characterized by a progressive myxomatous degeneration of valve leaflets and chordae tendineae [106, 110], differently from what occurs in chronic ischemic mitral regurgitation which is a secondary pathologic entity resulting from subvalvular remodeling and leaflet tethering induced by myocardial infarction with subsequent annulus dilatation and flattening. MVP represents a slowly developing process which usually shows a benign course, since less than 10% of valvular lesions progress to severe regurgitation requiring surgical treatment. In some occasions patients affected by worsening MR suffer from a variety of symptoms mostly represented by dyspnoea and palpitations related to secondary onset of supraventricular tachyarrhythmias, conditions that usually require repeated hospitalization and different diagnostic tests execution. Considering the wide diffusion of MVP, this creates high costs for health organizations, even if only a small portion of patients will need surgical therapy during their lifetime.

**Figure 9:**
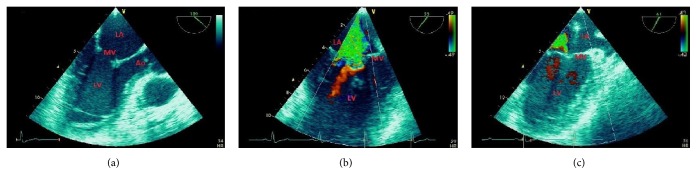
A 35-year-old female patient with systemic lupus erythematosus and progressive dyspnea from severe mitral regurgitation (MR). Patient had percutaneous mitral valve (MV) clip implantation. (a) Thickened MV leaflets. (b) Severe MR by TEE. (c) MR reduction after percutaneous MV clip implantation. Ao = aorta; LA = left atrium; LV = left ventricle.

It has become evident that moderate-to-severe MR, even in the absence of LV dilatation and dysfunction, may have adverse prognostic consequences. Thus, accurate echocardiographic quantification of MR is vitally important in clinical medicine, especially when planning surgery or interventional percutaneous procedures [9, 107] as the mitral clip implantation (Figure 9). Because of the mitral valve's structural complexity, MR is often difficult to define. Both qualitative and quantitative approaches are used. Color Doppler imaging allows measurement of the regurgitant jet area and vena contracta (VC) width; these two qualitative methods are simple to apply in daily practice but often are inaccurate, especially in patients with eccentric MR. Two-dimensional (2D) quantitative methods include the calculation of regurgitant fraction, regurgitant volume, and proximal isovelocity surface area. With three-dimensional (3D) echocardiography, many of the geometric assumptions necessary with 2D imaging are obviated, such as the depiction of the VC, which often is noncircular, and anatomic regurgitant orifice area, which usually is nonplanar.

Aortic valve stenosis (AS) represents a significant cause of morbidity and mortality [111]. The prevalence of calcific AS increases with age as it is around 5% in individuals aged 55 years and around 10% in individuals aged 80 years and older. The most common aetiology is the calcification of a normal trileaflet valve or a congenital bicuspid valve. Rheumatic aortic stenosis is less prevalent in the economically developing regions, although it remains a frequent cause of mortality in more economically challenged countries. The prognosis in patients with symptomatic aortic stenosis is poor, with the interval from the onset of symptoms to the time of death being ~2 years following heart failure, ~3 years following syncope, and ~5 years following angina.

**Figure 10:**
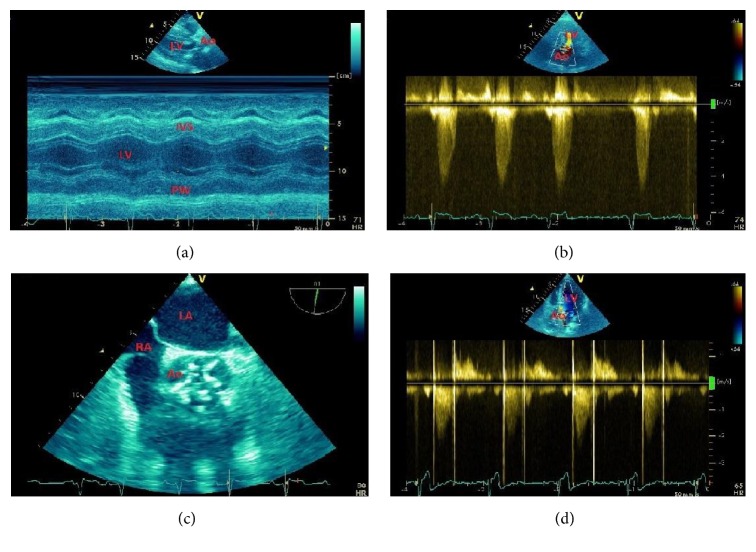
A 46-year-old male patient with valvar aortic stenosis (AS) and angina pectoris. Patient had aortic valve surgery. (a) Left ventricular hypertrophy from pressure afterload. (b) High transvalvular pressure gradient (81 mmHg peak, 44 mmHg mean) by color Doppler. (c) Reduced aortic valvar area (0.7 cm^2^) by TEE. (d) Aortic pressure gradient reduction (12 mmHg peak, 7 mmHg mean) after aortic valve surgery. Ao = aorta; IVS = interventricular septum; LA = left atrium; LV = left ventricle; PW = LV posterior wall; RA = right atrium.

Complex presentations with multiple comorbid conditions make often the diagnosis and management of AS challenging. Clinical signs and symptoms are of limited use in distinguishing critical from noncritical AS due to unsatisfactory sensitivity and specificity especially in the aged. Moreover, assessment of the symptomatic status and severity of valvular lesion can be disappointing because of the subjectivity of symptoms and ambiguity of individual functional capacity. The current guidelines approve two-dimensional transthoracic echocardiography (TTE) as the diagnostic test of choice for the assessment of AS. In addition to TTE, 2D transesophageal echocardiography (2DTEE) provides images with higher resolution (Figure 10), while real time three-dimensional TTE and 3DTEE facilitate spatial recognition of both anatomy and function and allow for the direct assessment of stenosis severity. Severe AS is conventionally defined as a peak aortic velocity > 4 m/s, a mean gradient > 40 mmHg, and a valve area < 1 cm^2^ [111]. Indications for aortic valve replacement are symptomatic severe AS, asymptomatic patients with severe AS and LV ejection fraction < 50%, and patients with severe AS undergoing cardiac surgery for other indications.

The majority of aortic valve replacements are surgical aortic valve replacement. However, in high-risk patient groups, factors such as increasing age, prior cardiac surgery, and other comorbidities, such as heart failure, respiratory, and renal diseases, are associated with a high potential for operative mortality and morbidity. Transcatheter aortic valve implantation (TAVI) was first performed in 2002 as a less invasive approach and is now recommended as an alternative strategy for patients in high risk surgical groups [112–114]. Multimodality imaging including echocardiography, computed tomography (CT), and CMR plays a pivotal role in the selection and planning process [113, 114]; however, echocardiography remains the primary imaging modality for patient selection, intraprocedural guidance, postprocedural assessment, and long-term follow-up.

## 4. Conclusions

Echocardiography using both conventional and more recent modalities is the primary diagnostic tool for the assessment of early cardiac dysfunction. It is a cost-effective technique and offers real-time imaging with high spatial and temporal resolution. Three-dimensional echocardiography and two-dimensional and three-dimensional speckle tracking echocardiography may provide additional diagnostic information. It is plausible that echocardiography will further evolve in the future with improvements in technology providing better clinical help. A strong professional liaison between echocardiologists and occupational physicians aids the process of offering effective support and management for the cardiovascular patients in the workplace.
